# Advances in plant imaging across scales

**DOI:** 10.1002/aps3.11550

**Published:** 2023-10-18

**Authors:** Pamela S. Soltis, Luiza Teixeira‐Costa, Pierre Bonnet, R. Gil Nelson

**Affiliations:** ^1^ Florida Museum of Natural History University of Florida Gainesville Florida 32611 USA; ^2^ Biodiversity Institute University of Florida Gainesville Florida 32611 USA; ^3^ Vrije Universiteit Brussel Brussels Belgium; ^4^ CIRAD, UMR AMAP Montpellier France; ^5^ AMAP, Université de Montpellier, CIRAD, CNRS, INRA, IRD Montpellier France

New imaging technologies are dramatically transforming all of biology. From remote sensing of continents to computed tomography (CT) scanning of individual organisms or parts of organisms, novel views are emerging that span planetary to suborganismal scales. In plant biology, observations from satellites (e.g., Deneu et al., 2021; Cavender‐Bares et al., 2022) and airborne instruments (e.g., Sun et al., 2021) are providing new insight into the distribution of botanical diversity, species abundance, and ecosystem productivity and how these features are changing in response to human activity. At the same time, advances in X‐ray technologies are revealing exquisite anatomical detail of both living and fossil plant structures (Brodersen and Roddy, 2016). Innovations in imaging, largely enabled by the development of new sensors and analysis capabilities, are also capturing specific attributes of individual plants as well as their community context in the field.

In this special issue of *Applications in Plant Sciences* (*APPS*), we explore innovations in imaging and their contributions to plant biology. The 10 papers included in this collection span imaging of live plants in the field to chemical mapping of specific compounds. The authors emphasize sample preparation techniques, practical aspects of image capture, standardization of imaging techniques and resulting images, multiple forms of image analysis, and alternatives for image archival in public repositories. Moreover, the diversity of the imaging approaches and protocols presented in this collection can be applied to a broad range of research, teaching, and public outreach.

Two papers in this special issue note the lack of consistency in photographs of plants taken in the field. These photographs might serve as a virtual voucher of a rare species (when destructive sampling would be detrimental to the population) or as a source of plant traits for ecological or evolutionary research, but field photographs of plants are rarely standardized. Unlike other groups of organisms for which “standard views” have been developed, the vast diversity of plants in terms of both size and structure precludes many traditional approaches to standardization. These issues, as well as others, render currently available collections, such as those downloadable from iNaturalist (https://www.inaturalist.org/), less useful than they could be if images were captured, processed, and archived following specified standards. To standardize and improve the usefulness of field‐captured images of plants, Weaver and Smith (2023a) report the development and implementation of FieldPrism, a system of photogrammetric markers, QR codes, and software to automate the curation of snapshot vouchers. They also developed FieldStation, a mobile imaging system that records images, GPS location, and other metadata on multiple storage devices. The combined use of FieldPrism and FieldStation will facilitate the rapid and standardized capture of field‐based plant traits.

The application of a standard protocol for capturing field images can also facilitate downstream image analysis and modeling, allowing the creation of three‐dimensional (3D) models of plants. These models allow the digital preservation of the shape, size, and architecture of an organism, as these features would otherwise be lost when captured only via pressed specimens or two‐dimensional photographs. Thus, James et al. (2023) provide detailed protocols for capturing images of plant specimens in the field and producing 3D models from the images using photogrammetry, a modeling approach that has become increasingly popular in different areas of biodiversity research. To showcase the applicability of their customizable protocol, the authors consider specimens of six different species exhibiting a range of surface:volume proportions. Moreover, the authors provide a thorough list of all equipment used in the field for photographing the specimens.

Beyond individual specimens, the uses of digital imaging and photogrammetry methods are also explored by Tirrell et al. (2023) for their increasing value in integrating systematics, conservation, plant ecology, and the broader study of plant diversity. The authors propose and demonstrate the use of photogrammetry as a nondestructive protocol for critical long‐term monitoring of research plots while reducing the possibility for inadvertent damage to sensitive, difficult‐to‐access, unpermitted, or otherwise inaccessible plant communities. The photogrammetry and structure‐from‐motion methods they describe are low‐cost, efficient, less technical to implement than some other photogrammetric solutions, and allow for continued surveying efforts in areas where permanent structures or other surveying methods are not feasible. These methods will also allow users to accurately survey and record sensitive plant communities through time. Although the techniques described have been developed and tested largely in alpine landscapes, they are broadly applicable to a wide range of monitoring activities.

A fourth paper using field‐captured photographs focuses on the analysis of color using images available on iNaturalist. To allow the rapid generation of color data, Luong et al. (2023) present a computational pipeline developed using R scripts and showcasing the utility of R shiny apps for enhancing iNaturalist collections and aiding users, including students, in natural history research. As an example, the authors analyze variation in *Erysimum capitatum*, a native North American species that exhibits a wide range of flower colors. The pipeline they developed allowed the testing of interesting hypotheses related to color spatial autocorrelation, climate correlation, and elevational gradients. This work highlights the enormous potential of citizen/participatory science data sets to increase the breadth of sampling for scientific research. This new method of extracting color from non‐standardized photographs makes it possible to take advantage of the large quantities of multimedia data generated on flora. The work also reinforces the value of collaborations between ecologists, computer scientists, and citizen/participatory science networks in conducting research in ecology and plant evolution.

In a complement to these innovations regarding field‐captured photographs, two papers in this special issue deal with images of samples in herbarium or other research collections. Although seeds often carry valuable information about local environmental conditions and evolutionary history, scoring seed characters has remained tedious and time‐consuming. Moreover, non‐standardized imaging techniques have yielded inconsistent results that make it difficult to quantify and interpret variation in seed traits. In response to these impediments, Steinecke et al. (2023) report a standardized high‐throughput technique to record seed number, seed area, and seed color from a collection of images using a model that relates seed area to pixel count. Application of this approach to seeds of *Arabidopsis thaliana*, *Brassica rapa*, and *Mimulus guttatus* demonstrated high reliability in the measurement of seed traits, opening the door to future studies of seed traits and the ecological and evolutionary drivers that have shaped them.

The second paper addressing images from herbarium specimens, which is also the second contribution by Weaver and Smith (2023b), updates and expands on a machine learning tool designed to autonomously measure leaves from images of digitized herbarium specimens. The original iteration of this approach, LeafMachine, was published by Weaver et al. (2020) and was trained on 2685 specimens spanning 20 plant families. The expanded LeafMachine2 approach published in this issue included training on an impressive 494,766 manually prepared annotations from 5648 herbarium images representing 2663 species. This updated version used a set of plant component detection and segmentation algorithms to isolate not just individual leaves, but also petioles, fruits, flowers, wood samples, buds, and roots. With this ability to rapidly generate large amounts of trait data, LeafMachine2 will become a critical tool for scientists seeking to understand taxonomic and phylogenetic relationships, species distributions, phenological responses to climate change, collection bias, and species interactions.

Segmentation algorithms are also at the core of the paper by Wolcott et al. (2023), who provide a new application of X‐ray micro‐CT scanning to help solve a persistent puzzle in pollination biology. The authors focus on the minute flowers of one of the world's most economically important agricultural species, *Theobroma cacao* (cacao, Malvaceae), whose yields are pollinator‐limited. The reduced size of the flowers and their elaborate morphology appear to limit pollinator access and movement within the flowers. While several small insects have been suggested as cacao pollinators, there is still uncertainty about the species involved. To advance the identification of specific pollinator species, Wolcott and colleagues combine the scanning of both flowers and potential pollinators with digital segmentation and tridimensional morphometric analysis. Their results reveal the main bottleneck for pollinator access and identify different levels of likelihood for putative pollinators and floral reward microstructures. The methods described by the authors, including sample preparation protocols and detailed codes for geomorphometric analysis, can inspire the further incorporation of geometry and floral reward studies to strengthen plant–pollinator trait‐matching models for cacao and other species.

The study by Long et al. (2023) also describes advances in sample preparation, addressing the case of using matrix‐assisted laser desorption/ionization (MALDI) mass spectrometry imaging (MSI) for a variety of plant species. In this technique, which allows the spatial analysis of chemical distribution in a tissue, a laser beam is fired at a matrix‐coated sample, transferring energy to the molecules extracted from the tissue. These molecules are then resealed from the surface, ionized, and detected using mass spectrometry. As noted by the authors, each of these steps can present difficulties when analyzing plant samples. Thus, Long and collaborators provide a general procedure for the easy preparation of press‐dried samples for analysis by MALDI‐MSI without the need for freezing or cryosectioning. Their simple protocol covers all steps of sample preparation, from the drying, delipidation, and application of the MALDI matrix to the parameters used for data acquisition. By analyzing flowers and leaves of plants with a variety of polyphenolic compounds, the authors confirm the wide applicability of the proposed protocol.

A third paper dedicated to improving protocols and sample preparation is provided by Klahs et al. (2023) for maceration of soft plant tissues. While a wealth of maceration techniques have been described, most protocols employ hazardous chemicals, thus rendering such methods unsuitable for classrooms. To help solve this issue in a cost‐effective way, the authors propose a protocol using pectinase as the agent for disrupting the adhesion among the cells of plant tissues. The protocol is shown to be effective in macerating both fresh and herbarium‐sampled leaves of different species, including plants with thick cuticle, abundant trichomes, and latex. This method can potentially be applied to a wider variety of species than current methods allow and can be used in both research laboratories and classrooms.

Finally, also focusing on images obtained from leaf samples, Green and Losada (2023) developed an open‐source code suitable for high‐throughput automation for measuring the length of leaf veins per area. This measurement has become the standard for comparing leaves with different vein densities and exploring the diversity of patterns expressed by different species. Since its first use, many approaches have attempted to standardize, automate, and facilitate its recording. However, major disagreements remain and to date have not been resolved. In their contribution, the authors propose three alternative new methods for measuring vein density using image analysis, making it possible to improve on current approaches. Each of the solutions presented in this work, and explored on more than 230 angiosperm leaves, has distinct practical, statistical, and biological limitations and advantages. Furthermore, the authors highlight that progress toward a more complete understanding of leaf vein biology requires not only the adoption of improved techniques and the use of advances in microscopy and computational speed, but also a commitment to sharing the original imagery and open‐source analytical code generated by researchers.

Together, this collection of papers demonstrates some of the innovations in imaging and image analysis in the plant sciences, and we hope that it will stimulate further developments in both image capture and analysis. Connecting novel imaging approaches with machine learning and other AI methods, such as those reported in a previous special issue of *APPS* (“Machine Learning in Plant Biology”; June and July, 2020), is likely to yield even further advances of the spectacular imaging techniques and pipelines reported here.

## AUTHOR CONTRIBUTIONS

P.S.S. and R.G.N. initiated this special issue, and L.T.‐C. and P.B. contributed to its development. In addition to handling editorial duties for the manuscripts in this issue, all authors wrote portions of this article, made comments and suggestions to improve it, and approved the final version of the manuscript.

## References

[aps311550-bib-0001] Brodersen, C. R. , and A. B. Roddy . 2016. New frontiers in the three‐dimensional visualization of plant structure and function. American Journal of Botany 103(2): 184–188. 26865119 10.3732/ajb.1500532

[aps311550-bib-0002] Cavender‐Bares, J. , F. D. Schneider , M. J. Santos , A. Armstrong , A. Carnaval , K. M. Dahlin , L. Fatoyinbo , et al. 2022. Integrating remote sensing with ecology and evolution to advance biodiversity conservation. Nature Ecology and Evolution 6: 506–519. 35332280 10.1038/s41559-022-01702-5

[aps311550-bib-0003] Deneu, B. , M. Servajean , P. Bonnet , C. Botella , F. Munoz , and A. Joly . 2021. Convolutional neural networks improve species distribution modelling by capturing the spatial structure of the environment. PLoS Computational Biology 17(4): e1008856. 33872302 10.1371/journal.pcbi.1008856 PMC8084334

[aps311550-bib-0004] Green, W. A. , and J. M. Losada . 2023. How dense can you be? New automatic measures of vein density in angiosperm leaves. Applications in Plant Sciences 11(5): e11551. 10.1002/aps3.11551 PMC10617316 37915435

[aps311550-bib-0005] James, N. , A. Adkinson , and A. Mast . 2023. Rapid imaging in the field followed by photogrammetry digitally captures the otherwise lost dimensions of plant specimens. Applications in Plant Sciences 11(5): e11547. 10.1002/aps3.11547 PMC10617317 37915433

[aps311550-bib-0006] Klahs, P. C. , E. K. McMurchie , J. J. Nikkel , and L. G. Clark . 2023. A maceration technique for soft plant tissue without hazardous chemicals. Applications in Plant Sciences 11(5): e11543. 10.1002/aps3.11543 PMC10617305 37915428

[aps311550-bib-0007] Long, Z. G. , J. V. Le , B. B. Katz , B. G. Lopez , E. D. Tenenbaum , B. Semmling , R. J. Schmidt , et al. 2023. Spatially resolved detection of small molecules from press‐dried plant tissue using MALDI imaging. Applications in Plant Sciences 11(5): e11539. 10.1002/aps3.11539 PMC10617318 37915436

[aps311550-bib-0008] Luong, Y. , A. Gasca‐Herrera , T. M. Misiewicz , and B. E. Carter . 2023. A pipeline for the rapid collection of color data from photographs. Applications in Plant Sciences 11(5): e11546. 10.1002/aps3.11546 PMC10617320 37915431

[aps311550-bib-0009] Steinecke, C. , J. Lee , and J. Friedman . 2023. A standardized and efficient technique to estimate seed traits in plants with numerous small propagules. Applications in Plant Sciences 11(5): e11552. 10.1002/aps3.11552 PMC10617364 37915429

[aps311550-bib-0010] Sun, Z. , X. Wang , Z. Wang , L. Yang , Y. Xie , and Y. Huang . 2021. UAVs as remote sensing platforms in plant ecology: Review of applications and challenges. Journal of Plant Ecology 14(6): 1003–1023.

[aps311550-bib-0011] Tirrell, A. J. , A. E. Putnam , M. I. J. Cianchette , and J. L. Gill . 2023. Using photogrammetry to create virtual permanent plots in rare and threatened plant communities. Applications in Plant Sciences 11(5): e11534. 10.1002/aps3.11534 PMC10617319 37915437

[aps311550-bib-0012] Weaver, W. N. , and S. A. Smith . 2023a. FieldPrism: A system for creating snapshot vouchers from field images using photogrammetric markers and QR codes. Applications in Plant Sciences 11(5): e11545. 10.1002/aps3.11545 PMC10617303 37915427

[aps311550-bib-0013] Weaver, W. N. , and S. A. Smith . 2023b. From leaves to labels: Building modular machine learning networks for rapid herbarium specimen analysis with LeafMachine2. Applications in Plant Sciences 11(5): e11548. 10.1002/aps3.11548 PMC10617304 37915430

[aps311550-bib-0014] Weaver, W. N. , J. Ng , and R. G. Laport . 2020. LeafMachine: Using machine learning to automate leaf trait extraction from digitized herbarium specimens. Applications in Plant Sciences 8(6): e11367. 32626609 10.1002/aps3.11367 PMC7328653

[aps311550-bib-0015] Wolcott, K. A. , E. L. Stanley , O. A. Gutierrez , S. Wuchty , and B. A. Whitlock . 2023. 3D pollination biology using micro‐computed tomography and geometric morphometrics in *Theobroma cacao* . Applications in Plant Sciences 11(5): e11549. 10.1002/aps3.11549 PMC10617321 37915432

